# Perceptions and experiences of patients living with implantable cardioverter defibrillators: a systematic review and meta-synthesis

**DOI:** 10.1186/s12955-016-0561-0

**Published:** 2016-11-17

**Authors:** Sim Leng Ooi, Hong-Gu He, Yanhong Dong, Wenru Wang

**Affiliations:** 1 Alice Lee Centre for Nursing Studies, Yong Loo Lin School of Medicine, National University of Singapore, Level 2, Clinical Research Centre, Block MD 11,10 Medical Drive, Singapore, Singapore; 2 Department of Pharmacology, Yong Loo Lin School of Medicine, National University of Singapore, Singapore, Singapore

**Keywords:** Implantable cardioverter defibrillator, Perceptions, Experiences, Quality of life, Coping strategies, Leaning needs, Systematic review

## Abstract

**Background:**

Sudden cardiac deaths have become a growing major public health concern that affects the world. Despite the various etiologies, life-threatening cardiac arrhythmias attribute the main cause of sudden cardiac deaths. Therefore in certain groups of high-risk patients, the Implantable Cardioverter Defibrillator (ICD) has been recommended as either a secondary or primary prophylactic method of prevention.

**Objective:**

To summarise the perceptions of ICD recipients and provide an overview of their experiences with regards to the quality of life, coping strategies, and learning needs.

**Methods:**

A systematic search was conducted using CINAHL, MEDLINE, PsycINFO, Scopus, Cochrane Library, and Web of Science. Primary research articles published from January 2005 to January 2016 that met the inclusion criteria were selected and assessed for methodological quality.

**Results:**

Thirty-nine articles consisting of 16 qualitative studies, 22 quantitative studies, and 1 mixed methods study were included for the meta-synthesis. Findings extracted from these studies were grouped into eight subthemes with ‘living the ICD experience: a constant process of redefining oneself’ emerging as an over-arching theme.

**Conclusion:**

This review provides insight into the perspectives and experiences of ICD recipients. Current evidence highlights the need for healthcare professionals to improve future care standards and develop a patient-centric holistic program that meets the specific needs of ICD recipients. Moreover, future studies are required to address the research gaps identified and also explore the perceptions of patients living with ICD in the Asian context.

## Background

Sudden cardiac arrest describes as an abrupt state where the heart ceases to pump and causes the vital organs to be deprived of oxygen supply [1]. With a grim survival rate of less than 1% globally [2], most of these cases eventually result in unanticipated sudden cardiac deaths, generally within an hour of acute symptoms onset in people who may or may not have been diagnosed of any underlying pre-existing fatal cardiac conditions [3]. In fact, sudden cardiac deaths have become a growing major public health concern that affects the world [2, 3]. Life-threatening cardiac arrhythmias attribute to the main cause of sudden cardiac deaths [3, 4]. It is estimated that ventricular tachyarrhythmias annually account for approximately 6 million of the sudden cardiac death cases worldwide [2].

The Implantable Cardioverter Defibrillator (ICD) is a device that is surgically-inserted into patients’ chest for: (1) the constant monitoring and pacing of the heart rhythm; (2) anti-tachycardia pacing (ATP) which involves delivering a series of low-energy impulses to revert ventricular arrhythmias; and (3) defibrillation where a strong electrical shock is delivered to restore the heartbeats again [5, 6]. The ICD has been recommended as either a secondary prevention for survivors of prior ventricular tachycardia, ventricular fibrillation, and sudden cardiac arrest episodes or as a prophylactic primary prevention for patients with pre-existing cardiac conditions [1].

Since ICD implantation is effective in lowering the mortality rates of sudden cardiac deaths and prolonging the lifespan of patients with life-threatening cardiac conditions [7], it comes to a situation where ICD recipients will have to live with the device and their underlying chronic cardiac conditions for the rest of their lives. This systematic review aims to summarise the perceptions of ICD recipients and provide an overview of their experiences with regards to the quality of life, coping mechanisms, and learning needs. The review also hopes to identify the gaps in existing literature and healthcare practices. These findings will assist healthcare professionals in improving future care standards as well as developing a patient-centric holistic program that meets the specific needs of ICD recipients, thus improving their quality of life.

## Methods

### Search strategy

A systematic search was conducted in CINAHL, MEDLINE, PsycINFO, Scopus, Cochrane Library and Web of Science. Search terms including ‘implantable cardioverter defibrillator’, ‘ICD’, ‘automatic implantable cardioverter defibrillator’, ‘AICD’, ‘implantable defibrillator’, ‘perception’, ‘perspective’, ‘meaning’, ‘belief’, ‘attitude’, ‘experience’, ‘quality of life’, ‘psychosocial’, ‘psychological’, ‘physical’, ‘sexual’, ‘spiritual’, ‘patient education’, ‘knowledge’, ‘coping strategies’, and ‘support’ were used in various combinations in the search process according to the Boolean instruction and truncation notation [8]. The inclusion criteria were: (1) primary research journal articles published from January 2005 to January 2016; (2) English language publications; and (3) research that focused on the perceptions and experiences of adults living with ICD. The articles were excluded if they were: (1) editorials, commentaries, conference abstracts, opinion pieces, or review papers; and (2) focused solely on ICD technicalities, healthcare professionals, caregivers, adolescents, or children.

### Search outcomes and quality assessment

**Fig. 1:**
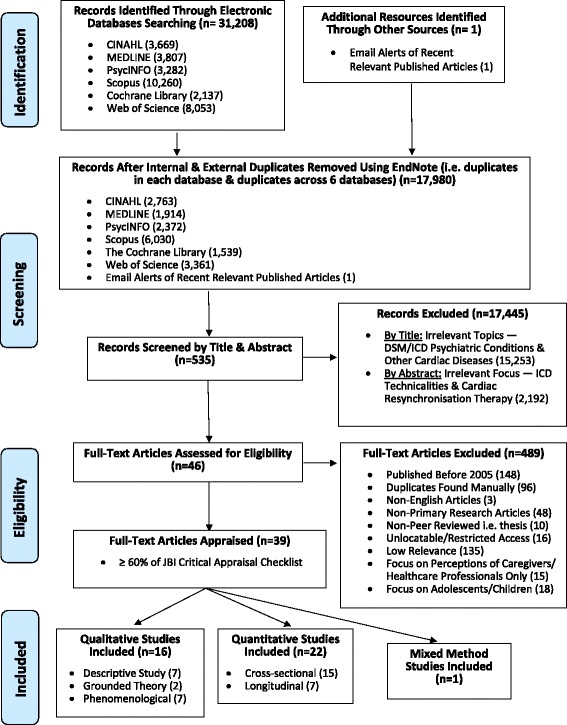
PRISMA Flowchart

The initial search across all databases yielded 31,208 articles. After removing the duplicates, the remaining 17,980 articles were screened for relevance based on the titles and abstracts. Subsequently, 535 full-text articles were retrieved, and after exclusion based on the inclusion criteria, the remaining 46 full-text articles were appraised for its quality. The Joanna Briggs Institute critical appraisal checklists [9] were used depending on the research designs to assess the methodological quality of the articles for its final inclusion in this systematic review. For the purpose of conducting a high-quality meta-synthesis, the articles were critically appraised by two independent researchers (SLO and WW). Of the 46 articles, only 39 articles met at least 60% of the appraisal checklists’ criteria on both independent assessments and were included in this review. The included studies are summarised in Table 1, and Fig. 1 depicts the PRISMA flowchart documenting the search process.

**Table 1:** Summary of included studies

Author (Year) Country	Research Aims	Research Design	Sample Characteristics	Outcome Measures	Instruments	Key Findings
Bilanovic et al. (2013) [37] Canada	Qualitative To explore experiences of phantom shocks in ICD recipients Quantitative To describe psychosocial correlates of objective and phantom shocks	Mixed Methods Qualitative Interpretive phenomenology Quantitative Cross-sectional descriptive correlational quantitative	Purposive sampling (17 participants) 9 ICD recipients with phantom shocks (PS) within the last 24 months - all males - mean age: 65.9 years 8 ICD recipients with objective shocks (OS) within the last 24 months - all males - mean age: 63.9 years	Qualitative Phantom shock experiences (8 participants, 1 refused to complete) Quantitative Psychosocial measurements of the level of: - Post-traumatic stress disorder (PTSD) - Depression & anxiety - Disease-specific distress - Social desirability	Qualitative Semi-structured interview (face-to-face) Quantitative Instruments: - PTSD Checklist – Civilian Version (PCL-C) - Hospital Anxiety & Depression Scale (HADS) - Cardiac Anxiety Questionnaire (CAQ) - Socially Desirable Response Set (SDRS-5)	Qualitative Theme 1: Phantom shock as a somatic experience PS is strikingly similar to OS; Vivid physical sensation of ‘punch in middle of breast’ Theme 2: Emotional impact of phantom shock Alarmed, confused, anxious, fear, helpless; Mistrust in ICD Theme 3: Searching for meaning Rationalize situation, trying to account for the cause of PS Quantitative - Both PS & OS ↑trauma & anxiety - PS ↑psychological distress (depression, PSTD) & social desirability - OS ↑heart-focused worry
Bolse et al. (2005) [15] United States	To describe the perceptions of ICD recipients on their life situations	Descriptive phenomenology (Dahlgren & Fallsberg’s approach)	Purposive sampling with maximum variation sampling (14 participants) - 8 males, 6 females - mean age: 55.71 years (range: 21–84 years) - average 2 years with ICD - 6 experienced shocks within the 1st year	Perceptions of life situations with ICD	Semi-structured interview (telephone call)	Category 1: Trust - Trust in ICD → Security & confidence for future Category 2: Adaptability - Adapt to limitations in life; Obligated to accept restrictions; Changing habits; Resume routine Category 3: Empowerment - Support from family & healthcare staff; Overprotection, felt dependent
Carroll and Hamilton (2005) [16] United States	To compare the QOL in those with ICD shock and those who did not receive shock during 1st year	Longitudinal, prospective, descriptive correlational quantitative	Convenience sampling (59 participants; Initially 81 participants – 84% retention rate) 16 Shock group - 13 males, 3 females - mean age: 57.5 years 43 Non-shock group - 29 males, 14 females - mean age: 64.8 years	Collected at two time points (at implantation & 1 year after): - Health status - Psychological distress - QOL Collected at one time point (1 year after): - Fear & concerns	Instruments: - Ferrans & Powers QOL Index - Medical Outcomes Study Short Form-36 (SF36) - Profile of Mood States (POMS) - Brodsky ICD Questionnaire	At 1 year, - Shock group significantly ↓mental health & vitality score than non-shock group - Shock group ↑anxiety, fatigue, psychological distress, & suffering than non-shock group
Carroll and Hamilton (2008) [45] United States	To investigate the changes in health status, QOL and psychological state following ICD implantation 4 years later	Longitudinal, prospective, descriptive correlational quantitative	Convenience sampling (41 participants; Initially 70 participants – 59% retention rate) - 30 males, 11 females - mean age: 60.4 years	Collected at six time points (at implantation, 6 months, 1 year, 2 years, 3 years, 4 years later): - Health status - Psychological distress - QOL	Instruments: - Quality of Life Index-Cardiac III (CQLI-3) - Medical Outcomes Study Short Form-36 (SF36) - Profile of Mood States (POMS)	- Mental health score improved ↑mental health & ↓psychological distress by 6 months post-ICD - Physical score worsened Physical sub-score significant ↑at 6 months but ↓functioning at 3 & 4 years - Fewer negative moods Total psychological distress score ↓significantly
Chair et al. (2011) [13] Hong Kong	To examine the HRQL and its relation with ICD shock-related anxiety and ICD acceptance	Cross-sectional, descriptive correlational quantitative	Purposive sampling (85 participants) - 65 males, 20 females - mean age: 59.7 years	Collected at one time point: - QOL - ICD shock-related anxiety - ICD acceptance	Instruments: - Chinese (Hong Kong) SF-12 Health Survey Standard Version 1.0 - Florida Patient Acceptance Scale (FPAS) - Florida Sock Anxiety Scale (FSAS)	- Physical component & mental component ↓than population norm - MCS (-) correlated with shock anxiety MCS (+) correlated with patient acceptance - Shock anxiety (-) correlated with patient acceptance - Age (+) associated with FPAS Age (-) related with FSAS - ICD shock (yes/no) does not but shock frequency (0, 1–2, ≥3) & gender significantly different on FSAS shock anxiety but not on MCS general mental functioning
Conelius (2015) [17] United States	To describe the experiences of women with ICD implantation	Descriptive phenomenology (Colaizzi’s approach)	Convenience sampling (3 participants) - all Caucasian women - age range: 34–50 years - average 1 year with ICD - none experienced shocks	Experiences of living with ICD	Unstructured interview (face-to-face)	Theme 1: Security blanket: If it keeps me alive, it’s worth it Sense of security **→** ↓Worry about medical emergencies Theme 2: A piece of cake: I do more than before Stable/↑QOL after post-op period; ICD implantation process was easy Theme 3: A constant reminder: I know it’s there Constant reminder of ICD by others and self; Affect body image Theme 4: Living on the edge: I do not want it to go off Fear of shock in public; Uncertainty over how it feels Theme 5: Catch 22: I’d rather not have it Rather not have but it’s medically necessary; No choice, had to adjust to ICD
Flanagan et al. (2010) [18] United States	To explore lived experiences of patients with 1–2 years post-ICD implantation	Descriptive phenomenology (Van Manen’s hermeneutic phenomenology approach)	Purposive sampling (14 participants) - 8 males, 6 females - median age: 55.7 years (range: 21–48 years) - 10 for secondary prevention - average 1–2 years with ICD - 6 experienced shocks in 1st post-op year	Experiences of patients 1–2 years after ICD implantation	Unstructured interview (telephone call)	Theme 1: Appreciation versus apprehension Gratitude; Anxiety over uncertainty of shock Theme 2: Maintaining structure & routine as a way to maintain sense of self Strong need to maintain structured routine; Reassure family that someone is checking on them Theme 3: Isolation & vulnerability Desire to connect with ICD patients but not attend support groups; Overwhelmed by isolation from family Theme 4: Being abandoned & still grieving Resistance to accept help & isolation → significant loss around time of illness (lost most important person); Still grieving Theme 5: Seeking advice, making decisions Many unanswered queries on sexual function & fear shocking partner/drive to avoid job loss/altered memory concerns
Flemme et al. (2005) [28] Sweden	- To describe theQOL and uncertainty in patients with ICD - To predict QOL at long-term follow-up	Longitudinal, descriptive correlational quantitative	Convenience sampling (35 participants; Initial 56 participants – 62.5% retention rate) - 23 males, 12 females - mean age: 58.7 years	Collected at four time points (pre-implantation, 1–10 months, 11–20months, ≥21 months average 6.9 years): - QOL - Uncertainty	Instruments: - Quality of Life Index – Cardiac version (QLI-C) - Mishel Uncertainty in Illness Scale Community (MUIS-C)	- Overall QOL & health/functioning remains unchanged over time; reasonably good at 6.9 years post-ICD - Socioeconomic & psychologic/spiritual domains ↓in 1st year - Baseline to long-term follow-up, family domain & uncertainty↓ - Uncertainty is a predictor of low QOL
Flemme et al. (2012) [27] Sweden	- To describe the coping strategies and coping effectiveness 6–24 months post-implantation - To explore the factors influencing coping strategies	Cross-sectional, descriptive correlational quantitative, multi-centred	Purposive sampling (147 participants; Initial 164 participants – 89% retention rate) - 121 males, 26 females - mean age: 63 years - 77 for secondary prevention - 38 experienced shocks	Collected at one time point: - Anxiety & depression - Perceived control - QOL - Coping strategies	Instruments: - Jalowiec Coping Scale-60 (JCS-60) - Quality of Life Index – Cardiac version (QLI-C) - Hospital Anxiety and Depression Scale (HADS) - Control Attitude Scale (CAS)	- Most seldom use coping strategies Coping strategies used perceived as fairly helpful - Perceive moderate control over condition - Optimism is the most frequently used Optimism is the most effective coping strategy - Anxiety & gender account for 26% of the variance in coping strategies - Female use more overall, optimistic, palliative & supportive coping - ↑Depression, ↑evasive coping ↑Perceived control, ↓fatalistic coping - Satisfied with QOL
Flemme et al. (2011) [28] Sweden	To explore the concerns of patients living with ICD (6–24 months) and how they handle daily their lives	Grounded theory (Constant comparative analysis)	Purposive sampling (16 participants; data saturation at 13 participants) - 9 males, 7 females - median age: 57.6 years (range: 31–78 years) - 12 for secondary prevention - average 6–24 months with ICD - 8 experienced shocks	Focus is not on acute phase near post-implantation: - Experiences in daily life (had ICD for 6–24 months) - Concerns - Management of concerns	Unstructured interview (face-to-face)	Core Category 1: Incorporating uncertainty in daily life Restricting activities (Strategies) Balance activity level with available resources → partly control life; Uncertain about activity level & type to prevent shock; Fear shock → restrictions & careful planning of activities of daily living (ADL) Distracting oneself Engage in other activity → ↓stress level, prevent thinking of negative aspects (denial & illusion) Accepting being an ICD recipient Accept – reality of condition/life situation (dependent on ICD & support from others but don’t mean accept helplessness)/body scar Re-evaluating life Reflective about life, changing values & expectations; Forced to live with uncertainty of future; Develop inner strength
Fluur et al. (2013) [11] Sweden	To describe the ICD recipients’ experiences regarding battery replacement and end-of-life issues	Descriptive qualitative	Quota sampling with maximum variation sampling (37 participants) - 23 males, 14 females - median age: 64 years (range: 29–88 years) - average 4.5 years with ICD - 21 for secondary prevention - 9 experienced shock - 8 with ICD replacement	Experiences with battery replacement & end-of-life issues	Semi-structured interview (face-to-face)	Theme 1: Being part of an uncertain illness trajectory Some had insight of their condition; some chose to ignore illness trajectory, live a day at a time Category 1: Standing at a crossroads Decision to replace ICD & when to discuss option The unreflecting way Replacing ICD a necessity; Offer protection from all causes of death; Adhere to doctor’s decision/ICD indication The deliberate choice Some disagreed with doctor’s advice to not replace, unless ICD no shock → unnecessary; Some are done with life Category 2: Progressing from one phase to another Anticipated preferences about ICD deactivation at end-stage Avoiding decisions The majority has no take on issue, difficulty talking about death; Unaware of deactivation option; Decide when the time come, live each day a time Choosing life at all costs Most kept it as long as possible, even with multiple shocks; Extend life; Misunderstanding of deactivation = immediate death/euthanasia Facing finality Some at end-stage reflected on mode of death; Few will make advance deactivation planning
Groeneveld et al. (2007) [19] United States	- To measure and compare the QOL among primary & secondary prevention - To identify the predictive factors for QOL in each group	Cross-sectional, descriptive correlational quantitative	Purposive sampling (120 participants) 45 Primary prevention group - 28 males, 17 females - mean age: 58 years 75 Secondary prevention group - 60 males, 15 females - mean age: 61 years	Collected at one time point: - QOL - ICD concerns	Instruments: - Euro-QOL-5D (EQ-5D), Visual Analogue Scale (EQ-VAS), - Health Utilities Index-Mark 3 HUI-3) - Medical Outcomes Questionnaires Survey Short Form-12 (SF-12) - Florida Patient Acceptance Survey (FPAS)	- No significant difference between both groups in all QOL scales - Both groups view their devices favourably according to the FPAS scale, no significant difference - Anxiety/concerns on: Lifting (40%) Sexual activity (19%) Driving (14%)
Habibovic et al. (2011) [33] Netherlands	To examine the effect of gender versus NYHA Class III/IV, ICD shock and Type D personality as determinantof anxiety & QOL using Cohen’s effect size estimates	Longitudinal, descriptive correlational quantitative, multi-centred	Purposive sampling (718 Participants; Initial 1080 participants – 66% retention rate) 139 Female Group - mean age: 58.3 years 579 Male Group - mean age: 61.4 years	Collected at two time points (at implantation & 12 months after): - Anxiety - QOL	Instruments: - Medical Outcomes Study Short Form-36 (SF36) - Spielberger’s State Trait Anxiety Inventory (STAI) - Type D Scale (DS14)	- No difference between men & women on mean anxiety scores - QOL difference in 2 out of 8 subscales of SF-36, women poorer physical functioning & vitality than men - In anxiety, effect size at baseline & 12 months for gender, NYHA class & ICD shocks → small Type D personality → large - In QOL, effect size at baseline & 12 months, Gender → small NYHA class & Type D personality → moderate to large
Herman et al. (2013) [50] Prag	To gain insight into patients’ opinions, attitudes and wishes regarding end-of-life decisions, ICD deactivation and their knowledge	Cross-sectional, descriptive quantitative	Convenience sampling (109 participants; Initial 112 participants, 3 excluded due to incomplete questionnaire) - 91 males, 18 females - mean age: 67.6 years - average 662.4 days with ICD	Collected at one time point: Survey questionnaire on end-of-life decisions, ICD deactivation & overall understanding	Instruments: - Self-developed survey questionnaire (qualitative questions – yes/no; quantitative questions – VAS)	- Felt safer with ICD (90.8%) - Health status improved (60.6%) - Discussed topic with doctor (7.3%) - Never thought of ICD deactivation at end-of-life (45.9%) - Wanted more information (40.1%) - Refused additional information on the issue (25.7%) 41.7% from secondary prevention & 22.4% from primary prevention refused to speak of deactivation - Deactivation a personal decision, won’t involve relatives (50.1%)
Humphreys et al. (2016) [42] United Kingdom	- To explore the perceived concerns and benefits of ICD - To explore the emotional responses to ICD and coping	Descriptive qualitative	Purposive sampling (18 participants) - 11 males, 7 females - range 30–68 years - 5 shock (1 out of 5 female) - 13 non-shock (6 out of 11 female) - 7 for secondary prevention - all except 1 had ≤1 year with ICD	Emotions, concerns and coping of ICD recipients	Semi-structured interview (face-to-face)	Theme 1: Physical consequences Physically aware of device in body → reminds of disease; Physical encumbrance – (1) Larger size (2) Protrusion (3) Arm adjacent to implant painful, restricted movement Theme 2: Emotional consequences Vulnerable/Uncertain (Non-shock patients with sudden cardiac arrests (SCA) episodes) Traumatized; ↑awareness of fine line between life and death; Changed perspectives to appreciate life and work Anxiety of receiving shocks Fear of 1st shock & its feelings (in non-shock patients) – Male: Focus on medical implications of shocks, Female: Focus on pain & failure to attend work Depression Loss of confidence – (1) Inability to resume work (2) Disappoint employers & unable to support spouse → loss of status & male role (3) ↓financial security; Loss of independence; Loss of physical fitness Theme 3: Coping with the ICD Avoidant/restriction; Acceptance – (1) resigned acceptance (no choice) (2) Grateful acceptance; Set goals for ‘new self’
Jacq et al. (2009) [46] France	To assess the point prevalence & severity of anxiety, depression & QOL using standardized scales on shock and non-shock patients	Cross-sectional, descriptive correlational quantitative	Purposive sampling (65 participants) 40 Shock group - 35 males, 5 females - mean age: 60.18 years - average 37.44 months with ICD - average 7.8 shocks 25 Non-shock group - 21 males, 4 females - mean age: 59.40 years - average 17.88 months with ICD	Collected at one time point: - Anxiety & depression - Health status	Instruments: - Medical Outcomes Study Short Form-36 (SF36) - Mini International Neuropsychiatric Interview according to DSM-IV (MINI) - Hospital Anxiety and Depression Scale (HADS)	- ↑Point prevalence of anxiety disorders in shock group (MINI shock: 37.5%, non-shock: 8%) - ↑Depressive symptoms in shock group but point difference of depressive disorders is insignificant - (+) correlation between the number of shocks & depressive symptoms - (-) correlation between the number of shocks & SF-36 mental health sub-score
Johansson and Strömberg (2010) [12] Sweden	To describe the perceptions of ICD recipients regarding driving & driving restrictions	Descriptive phenomenology (Dahlgren and Fallsberg’s approach)	Strategic theoretical sampling (20 participants) - 14 males, 6 females - Range: 43–82 years - 16 for secondary prevention - all had driving license – 16 driving & 4 ongoing restrictions	Perceptions of driving & driving restrictions	Unstructured interview (face-to-face)	Category 1: Achieving adherence to driving restriction Non-adherence when beliefs & preferences unaddressed/information unclear/given at inappropriate time Information needs Stress pre-implantation → less receptive to information; Lack discussion of consequences; Inconsistent information Individual interpretations Interpreted restriction as recommendation; Difficulty adapting – Driving whole life/2° prevention ban ~3 months; Blame restriction rather than condition Willingness to adapt Mutual understanding – Understood rationale, don’t think they are suitable/honour doctor’s agreement; Anxious of unable to do things as usual Category 2: Emotional influence of driving restrictions Wanted to keep driving privileges Loss of independence Losses – Social life changes/↓Independence/↓freedom → rely on others for ADL (felt useless/burden others/guilt)/limited; Changed self-image Perceived as physically-disabled; Less valuable; Lose personal identity; Altered self-image (dignity & self-respect) Category 3: Altered views on driving Importance of network Family support → driven around; (+/-) Comfort receiving help Influence on driving behaviour Change driving pattern – avoid driving/partner drive/avoid heavy traffic/limit time & distance Future perspectives Anxiety of causing accident, unsuitable driver; Unwilling to check for arrhythmia as fear license revoked
Mert et al. (2012) [38] Turkey	To describe the experiences of patients with ICD	Descriptive qualitative using focus group interview	Purposive sampling (19 participants) - 15 males, 4 females - mean age: 53.5 years - average 15.4 months with ICD - 13 experienced shock	Living with ICD: - Attitudes - Feelings - Beliefs - Reactions - Experiences	Semi-structured interview guide (focus group)	Theme 1: Experiences in the regular activities of daily life Restrict physical activity/quarrel/physical contact/shower alone → fear shock/ICD dislocation Theme 2: Experiences related to social life Cannot resume previous social activity; Cannot leave home → cellular phone phobia; Quit/change job Theme 3: Familial relationships ↓Sexual activity, partner uncomfortable; Overprotection Theme 4: Emotional changes Fear, nervous, anxiety (shock > no shock), anger; Uncertainty over shock timing Theme 5: Experiences related to ICD shocks Prior shock symptoms; ‘Blow on chest’; Anxiety, fear of death, helplessness (multiple shocks more pain) Theme 6: Patients’ experiences relating to receiving information/counselling from healthcare providers Inadequate information on impacts & shock management; Advised on driving & conditions affecting ICD; No chance to clarify doubts; Contradictory information received
McDonough (2009) [20] United States	- To describe the everyday experiences of younger adults (18–40 years) with ICD - To describe the physiological and psychosocial issues of younger adults - To identify the coping strategies	Descriptive qualitative	Purposive sampling with maximum variation sampling (20 participants) - Young adults age 18–40 years 14 Internet group - 6 males, 8 females - mean age: 32.9 years - average 4.1 years with ICD - 6 experienced shock 6 Telephone group - 2 males, 4 females - mean age: 35.2 years - average 3.4 years with ICD - 3 experienced shock	- Experiences of living with ICD - Physiological & psychosocial impacts of ICD - Coping strategies	Semi-structured interview Two methods of triangulation: - Internet group via website (written interview, email correspondence) - Telephone group via phone call (telephone interview)	Theme 1: A cautious transition to a new normal Initial diagnosis: Anxiety and concern Anxiety; Body image concerns; Anger with self; Resentment towards ICD; Depression Caution, awareness and security: Daily life with ICD Cautious; Security, trust, comfort in ICD Childbearing: Passing my disease to my children Concern of heredity cardiac conditions; Family planning – No kids/not more; Existing children – genetic testing/future preparations for ICD Financial concerns Out-of-pocket expenses; ↑Insurance premium; ICD & battery replacement costs; Job instability Physiological and psychosocial Physical restrictions; Pain, itching, scarring → embarrassment; Shock-related pain (female > male); Fear of shock in public; Body image & sexual concerns Strategies to manage life with an ICD: Be positive and live life to the fullest Positive; Adhere body cues; Healthy lifestyles; Online & social support; Educate others; Future planning
Morken, et al. (2010) [30] Norway	To explore the experience of living with ICD in daily life and the long-term (a minimum of 10 months)	Grounded theory (Strauss & Corbin’s approach)	Purposive sampling With maximum variation sampling (16 participants) - mean age: 54 years (range: 25–80 years) - average 4.5 years with ICD	Experiences of living with ICD: - Daily life - Long-term	Unstructured interview (face-to-face)	Core Category 1: Reconstructing the unpredictability of living with an ICD Category 1: Losing control (After shock) Uncertainty associated with the triggering of the device No pre-physical symptoms of arrhythmia; Unpredictability → depressing; ‘Struck by lightning’ Influence on the relationship with one’s partner Afraid to be alone; Dependent on partner Reduced physical activity ↓Physical activity to avoid shock/fear losing driving license for work → ↓well-being & sex life; Uncertainty over acceptable activity level; Most engage moderate daily exercise Category 2: Regaining control Being normal Resume normal life & perceive life good (no new shock) Learning to trust the ICD as a life saver Shock → remind death & show device functioned; Lifesaver; Grateful for new chance Category 3: Lacking support Lack of continuity & appropriate support from healthcare professionals Insufficient information on impacts & shock; Follow-up with different doctors; Consultation time limited Category 4: Seeking support Managing emotions Empathy in listening to their feelings Seeking guidance about physical activity Inactive from physical discomfort
Morken et al. (2014) [31] Norway	- To investigate the extent of shock anxiety & perceived support from healthcare professionals are related to PTSD symptoms - To examine the extent of perceived support from healthcare professionals moderate relationship between shock anxiety & PTSD symptoms	Cross-sectional, descriptive correlational quantitative	Purposive sampling (167 participants) - 133 males, 34 females - mean age: 64.4 years - 106 for secondary prevention - average 57 experienced shocks	Collected at one time point: - PTSD - Shock anxiety - Social support from healthcare professionals	Instruments: - Impact of Event Scale-Revised (IES-R) - Florida Sock Anxiety Scale (FSAS) - Patient Questionnaire on Empowerment	- Agree a little/strongly on constructive support (68.8%) Agree a little on non-constructive support (12%) - Experience moderate to severe PTSD symptoms (10–15%) - Associations between shock anxiety & PTSD symptoms significantly moderated by perceived non-constructive support from healthcare professionals ↑Non-constructive support, ↑tendency for PTSD especially those with shock anxiety
Morken et al. (2014) [32] Norway	To investigate the extent of perceived support from healthcare professionals and shock anxiety is related to device acceptance among ICD recipients	Cross-sectional, descriptive correlational quantitative	Purposive sampling (167 participants) - 133 males, 34 females - mean age: 64.4 years - 106 for secondary prevention - average 57 experienced shocks	Collected at one time point: - ICD acceptance - Shock anxiety - Social support from healthcare professionals	Instruments: - Florida Patient Acceptance Scale (FPAS) - Florida Sock Anxiety Scale (FSAS) - Patient Questionnaire on Empowerment	- Experience high device acceptance (84.4%) Experience device-related distress (4.8%) - Constructive support from healthcare professionals ↑device acceptance & moderate (-) relationship between shock anxiety & device acceptance → prevent shock anxiety leading to poor device acceptance Non-constructive support can ↓device acceptance
Myers and James (2008) [21] United States	To examine the differences in ICD indicators, anxiety and social support between ICD recipients who seek support group and those without	Cross-sectional, descriptive comparative quantitative	Convenience sampling (150 participants) 73 Support Attendees group - 55 males, 18 females - mean age: 67.71 years 77 Support Non-Attendees group - 65 males, 12 females - mean age: 68.38 years	Collected at one time point: - Anxiety - Social support & social network	Instruments: - Spielberger’s State Trait Anxiety Inventory (STAI) - Sarason’s 6-item Social Support Questionnaire (SSQ)	- Support attendees higher trait anxiety than non-attendees Support attendees less satisfied with social support than non-attendees - Trait anxiety higher in those diagnosed with tachycardia ↑Satisfaction with support, ↓trait & state anxiety - ↑Social network, ↓trait & state anxiety ↑Social network, ↑support satisfaction
Palacios- Ceña et al. (2011) [47] Spain	To determine the experience of Spanish male ICD recipients	Descriptive phenomenology (Giorgi approach)	Phase 1: Purposive sampling Phase 2: Theoretical sampling (22 participants, data saturation at 16) - men above age 18 years - average 44 months with ICD - 17 for secondary prevention - 10 experienced shocks	Experiences of living with ICD	Phase 1: Unstructured interview to not condition or guide participant (face-to-face) Phase 2: Semi-structured interview to elicit response on specific topics of interest (face-to-face) - Field notes - 12 personal letters - 4 diary extracts	Theme 1: Accepting the change ‘Changes (improves/restricts) in mobility & loss of independence’; ‘Changes in family & work status as advised to stop work’ – viewed (+) by senior positions/(-) by young & lower paying jobs Theme 2: Developing strategies (To adapt to ICD/Illness) ‘Avoidance & evasiveness’ of ICD-related events, avoid contact & stay indoors; ‘Search for alternative information’ Theme 3: Rethinking their relationship with their partner & becoming emotionally more distant ‘Importance of wife’; ↓‘Frequency & length of sexual relations’, fear of harming partner → emotionally-distant Theme 4: Giving up some of their independence Family support; Overprotection → lose independence but tolerated Theme 5: Transformed Reflection on life, changes in outlook & priorities; ‘Internal change’ in work, relationship & living Theme 6: With life insurance Love-hate attitude towards ICD Theme 7: Continual uncertainty & waiting ‘Discharge reminds that heart is deteriorating’; Waiting for discharges → uncertainty poorly-tolerated
Palacios- Ceña et al. (2011) [43] Spain	To explore the experience of elderly Spanish men with ICD implantation	Descriptive phenomenology (Giorgi’s approach)	- Purposive sampling - Snowball sampling (20 participants; Data saturation at 15 participants +5 participants for validation) - Elderly men age 71–83 years - average 52.7 months with ICD - 15 for secondary prevention - 13 experienced shocks/storm shocks	Experiences of living with ICD	Unstructured interview (face-to-face) - Field notes - 6 personal letters - 1 diary	Theme 1: Accepting changes Limited functional capacity & autonomy from fear of shocks → ADL changes Theme 2: Developing strategies to adapt to changes arising in all areas of the recipient’s life Hide health & ICD-related information; Confidence in healthcare staff, never seek other information sources; Positive attitude Theme 3: Living with someone Love & support from family; Strengthen couple’s relationship; Worry about family & try to stop them from being around Theme 4: Feel transformed Reflection on meaning of life & desire to live in peace; ‘Waiting’ for the end; Resignation/predestination; New life outlook & priorities before it’s too late Theme 5: Live feeling safe ICD as protector & lifesaver; Expectation of future shocks **→** uncertainty
Pedersen et al. (2013) [34] Netherlands	- To examine patients’ knowledge and willingness for information - To identify the prevalence and correlates of favourable attitude towards deactivation	Cross-sectional, descriptive correlational quantitative	Convenience sampling (294 participants stratified into 3 groups) - 110 Group 1: De novo implanted - 107 Group 2: Moderate experience - 77 Group 3: Considerable experience	Collected at one time point: - Patient’s knowledge about deactivation - Wishes for information	Instruments: - Self-developed survey questionnaire (qualitative questions – yes/no) - Generalised Anxiety Disorder Scale - Patient Health Questionnaire - Type D Scale	- Most are aware ICD deactivation option (68%, 1/3 unaware) - Important to inform patient of possibility (95%) - Discussion of deactivation issues ↑anxiety (82%) - When should discussion take place? (multiple responses): Before implantation (49%) During the dying process (26%) Battery replacement (17%) ↓Life expectancy (55%) - Made the decision for/against deactivation (246/84%) In favour of deactivation (195/79%) - ‘Wish for a worthy death – avoidance of shocks during dying’ independently associated with favourable attitude towards deactivation
Raphael et al. (2011) [49] England	To examine when end-of-life & device deactivation issues should be discussed and how much patients wish to know at pre-implantation	Cross-sectional, descriptive quantitative	Purposive sampling (54 participants) 29 Group 1: No shock group - 20 males, 9 females - mean age: 71 years - average 3.6 years with ICD - 18 for secondary prevention 25 Group 2: Shock group - 23 males, 2 females - mean age: 74 years old - average 3.3 years with ICD - 10 for secondary prevention	Collected at one time point: - When end-of-life & device deactivation should be discussed - How much patients wish to know at pre-implantation Additional questions for Group 2 regarding deactivation & factors influencing decision	Instruments: - Self-developed survey questionnaire (qualitative & quantitative questions)	- Poor understanding of ICD function Aware that ICD can be deactivated without being explanted (38%) - Want to be involved in deactivation decision (84%) All willing to address end-of-life issues, none found discussion distressing - Never considered ICD deactivation (87%) - When should discussion take place? Prior implantation (52%) Really ill (24%) - Situations to consider deactivation: Acutely unwell (82%) Frequency of shocks (70%) - Factors influencing deactivation decision: Prognosis (85%) ‘Quick death’ (70%)
Saito et al. (2012) [14] Japan	- To describe the experiences of living with arrhythmia & ICD - To evaluate their post- implantation experiences regarding insights on obtaining appropriate care for their conditions	Descriptive qualitative	No sampling method specified (22 participants) - 20 males, 2 females - mean age: 61.2 years old, (range: 35–79 years) - average 14 months with ICD - 8 experienced shocks	Experiences of living with arrhythmia & ICD	Semi-structured interview (face-to-face)	Category 1: Bewilderment stemming from arrhythmia & ICD implant Uncertainty about one’s own body Uncertainty about fatal arrhythmia & necessity of ICD Fear of arrhythmia ending my life Anxiety related to ICD shock (without shock – anxious of unknown, with shock – anxious of recurrence) Dissatisfaction with unforeseen results of ICD Dissatisfaction regarding limitations of ICD & lifestyle restraints; Discomfort of having foreign object Category 2: Facing reality of arrhythmia, the ICD & being able to continue life Confirming & managing lifestyle activities Permissible range of safe lifestyle activity; Concern on evaluating expansion of lifestyle activity Facing reality of the ICD & being able to continue life Objectification of themselves as being kept alive by machine Category 3: Giving meaning to living with arrhythmia & ICD Giving meaning to one’s illness Giving meaning to the value of ICD; Coming to terms with own lifestyle, acceptance Recognition of one’s disease Objectification of disease (gaining knowledge & new outlook); Return to original lifestyle despite changes in ADLs
Salmoirago-Blotcher et al. (2012) [22] United States	To evaluate if better spiritual well-being is associated with lower psychological distress in ICD outpatients	Cross-sectional, descriptive correlational quantitative	Convenience sampling (46 participants) - 32 males, 14 females - mean age: 65 years	Collected at one time point: - Psychological distress - Spiritual well-being	Instruments: - Hospital Anxiety and Depression Scale (HADS) - Functional Assessment of Chronic Illness Therapy-Spiritual Well-being (FACIT-SWB)	- ↑HADS, ↓FACIT-SWB - Spiritual well-being is independently associated with ↓psychological distress in ICD outpatients Spiritual well-being could be a protective factor against psychological distress in these high-risk patients
Spindler et al. (2009) [39] Denmark	- To examine if women are at greater risk of increased anxiety, depression, ICD concerns and lower device acceptance - To examine if women have poorer QOL than men after adjusted for demographic and clinical factors	Cross-sectional, descriptive correlational quantitative	Convenience sampling (535 participants) 97 Female Group - mean age: 55.22 years 438 Male Group - mean age: 62.94 years	Collected at one time point: - Anxiety & depression - QOL - ICD concerns - ICD acceptance	Instruments: - Hospital Anxiety and Depression Scale (HADS) - ICD Concerns Questionnaire (ICDC) - Florida Patient Acceptance4 Survey (FPAS) - Medical Outcomes Study Short Form-36 (SF36)	- Women ↑anxiety than men Women ↑ICD concerns than men Differences in depression insignificant - ICD patients with shocks ↑anxiety ICD patients with shocks ↑ICD concerns - Significant gender differences for 3 out of 8 subscales of SF-36 Women reporting poorer HRQL on all 3 subscales
Starrenburg et al. (2014) [35] Netherlands	To examine relationship between gender and patient-reported outcomes regarding general anxiety, device-related anxiety, depression and QOL	Longitudinal descriptive correlational quantitative	Purposive sampling (300 participants) 53 Female group - mean age: 59.8 years 247 Male group - mean age: 62.9 years	Collected at 5 time points (pre-implant, 2mths, 5mths, 8mths, 12mths): - Anxiety & depression - Health-related quality of life (HRQOL) - Shock-related anxiety - ICD acceptance	Instruments: - Hospital Anxiety and Depression Scale (HADS) - Florida Shock Anxiety Scale (FSAS) - Florida Patient Acceptance4 Survey (FPAS) - Medical Outcomes Study Short Form-36 (SF-36)	- Women has higher anxiety & shock-related anxiety than men within a year - On most HRQOL subscales, no gender differences except in physical functioning where women reported more improvement than men - Gender is independently associated with poorer device-related acceptance - Women expressing higher levels of concerns about body image than men
Steinke et al. (2005) [23] United States	To explore the sexual activity of patients & their partners post-ICD implantation	Descriptive qualitative Participants recruited from part of a larger quantitative study examining sexual issues & concerns from a diverse of samples of 2 support groups	Convenience sampling (12 participants) ICD Patients - 10 males, 2 females - mean age: 62 years - average 5.3 years with ICD - all except 1 sexually active – cease all sexual activity due to ICD discharge - 5 experienced ICD discharge during sexual activity Partners - 1 male, 3 females - mean age: 47 years	Post-ICD experiences: - ICD impacts on relationship & sexual relationship - Effect of ICD discharges on sexual activity - Patient education & sexual counselling needs - Preferred patient education - Other sexual concerns	Semi-structured interview (face-to-face)	Theme 1: Anxiety & apprehension Concerns about resuming sex Partner overprotectiveness Attentiveness to patients’ needs Fear of ICD firing with sexual activity Fear & anxiety related to ↑heart rate → may signal impending shock; (-) past experiences; Change sexual frequency Theme 2: Varying interests & pattern of sexual activity Strong/↑sexual interest despite anxiety; Explore other ways of affection; ↓frequency; Backing off & waiting before resuming sex after ICD discharge Theme 3: Powerfulness of ICD discharge Patient – ‘thunder going off chest’; Partner – ‘bumping together hard’; ICD discharge unpredictable Theme 4: A need for information & sexual counselling Provider relationships Preference of sharing sexual issues with healthcare staff based on knowledge level; Some staff indifferent/uncomfortable Educational approaches ICD support member with knowledge & experience; Need for information – most prefer sexual information provided pre-discharge, reinforce advice, answer queries, individualized Information for sexual counselling Lack of information on resuming sex
Strömberg et al. (2014) [29] Sweden	- To describe the knowledge on ICD at the end-of-life in a large national cohort of ICD recipients - To explore patient-related factors associated with insufficient knowledge regarding role of ICD in end-of-life	Cross-sectional, descriptive correlational quantitative	Convenience sampling (3067 participants) - 2438 males, 629 females - mean age: 66 years - average 5 years since ICD implantation - 1957 for secondary prevention - 1056 experienced shock	Collected at one time point: - Knowledge about ethical aspects - Knowledge differences by age & gender - Impact of insufficient knowledge on deactivation/replacement attitudes	Instruments: - EuroQol-5 Dimension (EQ-5D) - Experiences, Attitudes & Knowledge of End-of-Life Issues in Implantable Cardioverter Defibrillator Patients (EOL-ICD) Questionnaire	- Few scored all correct in EOL-ICD (3%; mean score: 6.6/11) - Insufficient knowledge in EOL-ICD 25th percentile (29%) ~1/3 thought deactivation = euthanasia Only 1 in 10 wants deactivation during terminal illness - Insufficient knowledge is associated with greater indecisiveness to make decisions on ICD deactivation in end-of-life or make decision that may not achieve a high quality of end-of-life experience e.g. favour replacing ICD even in seriously-ill/advanced age, keeping shock even in end-stage terminal illness
Svanholm et al. (2015) [48] Denmark	To explore the experiences & thoughts of octogenarian with ICD/CRT-D with a battery replacement due	Descriptive phenomenology (Ricoeur’s reflective phenomenology & interpretive approach)	Purposive sampling (11 participants) - 9 males, 2 females - mean age: 82.8 years (range: octogenarians 80–86 years - mean year range of implantation: 2003 - 10 for secondary prevention	Experiences regarding: - Everyday life - Views on life & death issues - Decision making - Communication with healthcare professionals	Semi-structured interview (face-to-face)	Theme 1: Feeling safe with the ICD The ICD: A life keeper ICD is a necessity to prolong life; Understood ICD hinder natural death → refuse replacement The battery level is important Even with remote follow-up, appreciate going down to reassure battery level ICD shock – No problem None had fear of shock; Some unsure if had shock – misunderstood knowledge Theme 2: The physician is an authority Being trustful View physician role as treat actively → replace when battery low; Place lives in doctors’ hands, grateful & satisfied Feeling fine knowing nothing Surprised when told of possibility to deactivate ICD/Refuse replacement Criminal act to deactivate the ICD or refuse ICD replacement View as an illegal act for doctors
Thomas et al. (2009) [24] United States, Canada & New Zealand	- To evaluate the changes in depression, anxiety and social support in heart failure patients who implanted ICD in SCD-HeFT - To evaluate effects of ICD shocks on age and NYHA class on these changes	Longitudinal, descriptive correlational quantitative	Purposive sampling (22 participants; Initial 57 participants – 38% retention rate) - 47 males, 10 females - all NYHA Class II/III heart failure - mean age: 59.8 years - 12 experienced shock	Collected at five time points (Initial, 6, 12, 18, 24 months): - Depression - Anxiety - Social support	Instruments: - Beck Depression Inventory-2 (BDI-II) - Spielberger’s State Trait Anxiety Inventory (STAI) - Social Support Questionnaire-6 (SSQ-6)	- Depression ↓significantly overtime overall but ↑in those with ICD shocks - Anxiety higher in NYHA Class III than Class II, ↓in Class III but remained the same in Class II - Amount of social support (-) related to age Young, more social support Social support ↓significantly over time but young ↓more
Vazquez et al. (2008) [25] Australia & United States	To investigate the areas of adjustment across 3 age groups of women from multiple centres	Cross-sectional, descriptive correlational quantitative, multi-centred	Convenience sampling (88 participants) 30 Young women group - ≤50 years 25 Middle women group - 50–64 years 32 Old women group - ≥ 65 years - average 3.1 years since ICD implantation - 33% experienced shocks	Collected at one time point: - Shock anxiety - Death anxiety - Body image concerns	Instruments: - Florida Shock Anxiety Survey (FSAS) - Multi-dimensional Fear of Death Scale (MFODS) - Florida Patient Acceptance Survey (FPAS)	- Young women has higher rate of shock anxiety, death anxiety & body image concerns than middle & older women
Verkerk et al. (2015) [36] Netherlands	- To investigate the impact on QOL in 1st year after ICD implantation for primary prevention of SCD among young adults between 18 and 50 years - To compare the QOL scores with available population norms	Longitudinal, descriptive quantitative	Convenience sampling (35 participants) - 18 males, 17 females mean age: 36.7 years	Collected at four time points (pre-implantation, 2, 6, 12 months): - Depression - Anxiety - QOL	Instruments: - Centre for Epidemiologic Studies Depression Scale (CED-D) - Spielberger’s State Trait Anxiety Inventory (STAI) - Medical Outcomes Study Short Form-36 (SF36) - Self-designed questionnaire to explore impacts of receiving ICD	- 29% of patients’ pre-ICD depression score (CES-D) higher than cut-off score of 16. After 2, 6 & 12 months → 23, 9 & 13% respectively - 71% of patients pre-ICD anxiety score (STAI) higher than cut-off of 40 After 2, 6 & 12 months → 40, 32 & 34% respectively - QOL significantly ↓ at pre-implantation & 2 months but improved with time & is comparable with population norms at 6 & 12 months - Self-designed questionnaire 1: ICD… Feel protected against cardiogenic condition (87%) More negative than positive effects (11%) Worry of ICD firing when nobody is around (22%) Influences the way I dress (16%) Can no longer do the things I enjoy (19%) Lead a normal life like everyone else (52%) - Self-designed questionnaire 2: Cardiogenic condition & ICD therapy have… Negative influence on my professional career (34%) Important influence on decision for children (36%) - Of 29 patients with job at baseline: 28% had lost/changed their from their condition/ICD 17% temporarily can’t work 31% ↓working hours
Versteeg et al. (2010) [40] Germany	- To examine if female ICD patients report more psychological distress than male patients - To examine if somato-sensory amplification mediates this relationship	Cross-sectional, descriptive correlational quantitative	Convenience sampling (241 participants) 80 Female group - mean age: 55.04 years 161 Male group - mean age: 60.29 years	Collected at one time point: Instruments: - Psychological distress - Somatosensory amplification	Instruments: - Symptom Checklist-90 (SCL-90) - Somatosensory Amplification Scale (SSAS)	- Female has more anxiety, phobic anxiety, & somatic health complaints than men Female has higher somatosensory amplification score than men - Somatosensory amplification is associated with more anxiety, phobic anxiety, & somatic health complaints - Somatosensory amplification mediated the association between gender & three domains of psychological distress
Williams et al. (2007) [44] Australia	To explore the experiences, concerns & needs of ICD recipients and their caregivers	Descriptive qualitative	Purposive sampling (22 participants) Age range: 30–80 years 11 ICD recipients - 8 males, 3 females - number of years with ICD: 4 had less than 2 years, 5 had 2–3 years, 2 had more than 3 years 11 Caregivers	Experiences, concerns & needs of recipients and caregivers	Semi-structured interview (face-to-face)	Theme 1: Physical & psychological adjustments stage Physical difficulties; Psychological distress; Coping with reality of illness, uncertainty & insecurity of future – denial, avoidance of topic, & refusal to resume normal activities Theme 2: Acceptance stage – Getting on with life - ICD accepted, normal routine resume; Strong will power - Play it down to people/avoid discussion - Forget about ICD being there - Reframe interpretation of personal situation, others less fortunate; ICD support group - Reassess lifestyle, make changes

*QOL* quality of life, *ICD* implantable cardioverter defibrillator

### Data extraction and analysis

A data extraction form was used to extract information pertaining to the research aims, research designs, sampling methods, sample characteristics, outcome measures, data collection instruments, and key findings. For the extracted qualitative data, a meta-synthesis was used to integrate findings and offer a new interpretation across the reviewed articles. Findings from the quantitative studies were synthesised and presented in a narrative manner. A thematic analysis approach [10] was adopted for this systematic review. The studies were firstly read and familiarised before identifying for implicit and explicit codes across the text. Similar occurring codes captured in the study findings were then categorised.

## Results

### Characteristics of included studies

Among 39 studies included, there were 16 qualitative, 22 quantitative, and 1 mixed-methods. Purposive and convenience sampling were the most common methods, and only two studies used either quota or strategic theoretical sampling [11, 12]. With the exception of two Asian studies i.e. Japan and Hong Kong [13, 14], the majority of studies were conducted in the Western countries, more frequently in the United States [15–25], Sweden [11, 12, 26–29], Norway [30–32], and Netherlands [33–36].

All of the quantitative studies (*n* = 22) were of a descriptive correlational design, and the sample size ranged from 35 to 3067 participants, in which the largest study was conducted in collaboration with the Swedish ICD and Pacemaker Registry [29]. For qualitative studies, two studies adopted a grounded theory approach whereas the other 14 studies used phenomenological and descriptive designs. While most qualitative studies reported only themes relating to the perceptions of ICD recipients, there were two particular studies that also explored and compared the experiences of their partners or caregivers. By comparison, the mixed-method study consisted of both interpretive phenomenological and cross-sectional descriptive designs for a holistic understanding of the phantom shock experiences.

### Meta- synthesis of study findings

Findings extracted from the studies were grouped into eight subthemes, with the synthesised finding of ‘living the ICD experience: a constant process of redefining oneself’ emerging as an over-arching theme (Table 2).

**Table 2:** Synthesised finding

Synthesised finding	Categories	Findings (Themes captured in the qualitative & quantitative study)
Living the ICD Experience: A Constant Process of Redefining Oneself	Describing ICD Shocks	Qualitative Study
		Phantom shock as a somatic experience
		Experiences related to ICD shocks
		Powerfulness of ICD discharge
		A cautious transition to a new normal – Physiological and psychosocial
		Reconstructing the unpredictability of living with an ICD – Losing control (Uncertainty associated with the of the device)
	Experiencing Uncertainty & Psychological Distress	Qualitative Study
		Emotional impact of phantom shock
		Living on the edge: I do not want it to go off
		Appreciation versus apprehension
		Emotional influence of driving restrictions – Loss of independence
		Emotional changes
		A cautious transition to a new normal – Initial diagnosis: Anxiety and concern & physiological and psychosocial
		Reconstructing the unpredictability of living with an ICD – Losing control (Uncertainty associated with the triggering of the device)
		Living with an ICD is living while… continual uncertainty and waiting
		Bewilderment stemming from arrhythmia and ICD implant – Uncertainty about one’s own body & fear of arrhythmia ending my life
		Anxiety & apprehension – Fear of ICD firing with sexual activity
		Emotional consequences – Vulnerable/uncertain, anxiety, depression
		Quantitative Study
		PTSD, anxiety, depression, social desirability [37]
		QOL mental health score, mood states [45]
		QOL, mood states, ICD concerns for shock versus non-shock [16]
		QOL mental component, shock anxiety, and ICD acceptance [13]
		QOL, anxiety for men versus women [33]
		QOL, uncertainty [26]
		QOL, ICD concerns [39]
		QOL, anxiety, depression [46]
		QOL mental health subscale, anxiety, depression for men versus women [39]
		QOL, anxiety, body image [35]
		Depression, anxiety for shock versus non-shock [24]
		Shock anxiety, death anxiety, body image for young versus old women [25]
		QOL, depression, anxiety, impacts of ICD [36]
		Anxiety, somatosensory amplification for men versus women [40]
	Impacting Self-Identity, Self-Image & Self-Perception	Qualitative Study
		A constant reminder: I know it’s there
		Seeking advice, making decisions
		Emotional influence of driving restrictions – Loss of independence & changed self-image
		Emotional changes
		A cautious transition to a new normal – Initial diagnosis: Anxiety and concern & physiological and psychosocial
		Bewilderment stemming from arrhythmia and ICD implant – Dissatisfaction with unforeseen results of ICD
	Needing Support & Maintaining Relationships	Qualitative Study
		Empowerment – Receiving emotional and tangible layman support & informational and tangible professional support
		Isolation and vulnerability
		Being abandoned and still grieving
		Altered views on driving – Importance of network
		Experiences related to social life
		Familial relationships
		Reconstructing the unpredictability of living with an ICD – Losing control (Influence on the relationship with one’s partner)
		Reconstructing the unpredictability of living with an ICD – Lacking support (Lack of continuity and appropriate support from healthcare professionals)
		Reconstructing the unpredictability of living with an ICD – Seeking support (Managing emotions & seeking guidance about physical activity)
		Living with an ICD is living whilst… rethinking their relationship with their partner and becoming emotionally more distant
		Living with an ICD is living while… giving up some of their independence
		Living with someone
		Anxiety and apprehension – Partner overprotectiveness
		Quantitative Study
		ICD acceptance, shock anxiety, professional support [31]
		PTSD, shock anxiety, professional support [32]
		Anxiety, social support [21]
		Social support [24]
	Identifying Learning Needs	Qualitative Study
		Empowerment – Informational and tangible professional support
		Seeking advice, making decisions
		Achieving adherence to driving restrictions – Information needs
		Patients’ experiences relating to receiving information/counselling from healthcare providers
		A need for information and sexual counselling – Provider relationships, Educational approaches & information for sexual counselling
	Developing Coping Strategies	Qualitative Study
		Searching for meaning
		Incorporating uncertainty in daily life – Distracting oneself & re-evaluating life
		A cautious transition to a new normal – Strategies to manage life with an ICD: Be positive and live life to the fullest
		Living with an ICD is living while… developing strategies
		Living with an ICD is living while… transformed
		Developing strategies to adapt to changes arising in all areas of the recipient’s life
		Bewilderment stemming from arrhythmia and ICD implant – Dissatisfaction with unforeseen results of ICD
		Feel transformed
		Giving meaning to living with arrhythmia & ICD – Giving meaning to one’s illness & recognition of one’s disease
		Getting on with life – Positive interpreting
		Coping with the ICD
		Quantitative Study
		QOL, anxiety, depression, coping strategies [28]
		Spiritual well-being, anxiety, depression [22]
	Making Adjustments & Gaining Acceptance	Qualitative Study
		Adaptability – Handling restlessness, tackling restrictions, & managing daily living
		A piece of cake: I do more than before
		Catch 22: I’d rather not have it
		Maintaining structure & routine as a way to maintain sense of self
		Incorporating uncertainty in daily life – Restricting activities
		Incorporating uncertainty in daily life – Accepting being an ICD recipient
		Achieving adherence to driving restrictions – Individual interpretations & Willingness to adapt
		Altered views on driving – Influence on driving behaviour & Future perspectives
		Experiences in the regular activities of daily life
		A cautious transition to a new normal – Caution, awareness and security: Daily life with an ICD
		A cautious transition to a new normal – Childbearing: Passing my disease to my children & financial concerns
		Reconstructing the unpredictability of living with an ICD – Losing control (Reduced physical activity)
		Reconstructing the unpredictability of living with an ICD – Regaining control (Being normal)
		Living with an ICD is living while… accepting the change
		Accepting changes
		Facing reality of arrhythmia, the ICD, and being able to continue life – Confirming and managing lifestyle activities & facing reality of the ICD and being able to continue life
		Varying interests and pattern of sexual activity
		Getting on with life Lifestyle changes – Resuming normal activities, not thinking about ICD, lifestyle changes & risk taking
		Physical consequences
		Quantitative Study
		QOL physical health score [45]
		QOL physical component [13]
		QOL physical functioning for men versus women [33]
		QOL physical and social functioning subscale, anxiety, depression for men versus women [39]
		QOL physical functioning [35]
	Planning for the End	Qualitative Study
		Being part of an uncertain illness trajectory – Standing at a crossroads & progressing from one phase to another
		The physician is an authority – Feeling fine knowing nothing & criminal act to deactivate the ICD or refuse ICD replacement
		Quantitative Study
		ICD deactivation [34, 49, 50]
		ICD deactivation knowledge [29]

*QOL* quality of life, *ICD* implantable cardioverter defibrillator

#### Describing ICD shocks

The shock episodes experienced by participants can be classified into: (1) objective shocks, which refer to the actual shock therapies that were delivered and recorded by the ICD; and (2) phantom shocks, the phenomena where participants reported that sensations of shock were found to be unrecorded during ICD interrogations [37]. Comparing the participants’ accounts across several studies, both objective and phantom shock occurrences were found to be often abrupt and unexpected [23, 30, 37, 38]. This is because phantom shocks were predominantly encountered during sleep or sleep-wake transitions with rarer instances while awake [37]. By comparison, although some participants recalled experiencing physical symptoms of nausea, warmth, dizziness, and altered heart rhythm preceding objective shocks [20, 38], the majority were unable to foresee the impending shocks [38].

Consistent across several qualitative studies, participants used terms of high intensity to describe their physical and sensory experiences with objective shocks. Common terms consisting of ‘explosion’, ‘blow’, ‘bomb’, ‘shot by gun’ [20, 23, 30], or terms with close associations like ‘thunder’ [23], ‘lightning’ [30, 38], and even phrases of similar meanings like ‘electric shock’ [38] and ‘sticking your finger in the light socket’ [23] illustrated the suddenness, striking, and high impact nature of objective shocks. Partners in close body contact with the participants also reported feeling a sudden repulsive force of being ‘kicked’ or ‘thrown’ which corroborated with the participants’ account of experiencing objective shocks [23]. Accompanying these shocks, seeing light flashes [23, 38] were more commonly reported compared to hearing popping noises [23]. Participants with experiences of both objective and phantom shocks had described their intensity and characteristics to be vividly similar and indistinguishable [37]. However, upon closer examination, it was observed that the participants tend to use terms of comparatively lower intensity like ‘punch’ and ‘kick’ in their reference to phantom shocks [37].

Nevertheless, objective and phantom shocks were similar in that both physical sensations were felt mostly in the chest [23, 37, 38] and pain was also recounted in the aftermath [23, 37, 38]. Specifically in objective shocks, pain experiences varied widely. With the majority reporting mild discomfort [20] to those experiencing multiple shocks having greater pain [38] and females describing more intense pain reaction than males [20]. In several studies, it was found that females tend to have greater anxiety than males [35, 39, 40] and anxiety could have potentially exaggerated their pain experience as explained by the nocebo hyperalgesia phenomenon [41]. Post-shock symptoms like nausea and dizziness were also reported in objective shocks [15, 20].

#### Experiencing uncertainty and psychological distress

In the initial post-ICD implantation period, participants experienced feelings of anxiety, fear, depression, helplessness, anger, insecurity, and uncertainty [14, 17, 18, 20, 23, 30, 38, 42–44]. These negative emotions described in the qualitative interviews concurred with quantitative findings on poorer psychological well-being in the early phase [24, 28, 36, 45]. Among them, fear and anxiety were the most prevalent emotions following post-discharge [20].

The majority were anxious over the unpredictability and occurrence of shocks as well as the potential loss of independence with ICD [14, 18, 20, 30, 38, 42]. There were four quantitative studies that explored different anxiety levels between genders. Despite the differences in geographical locations and anxiety instruments, three studies reported higher anxiety levels in females than males [35, 39, 40]. Versteeg et al. [40] first established that somatosensory amplification could have mediated the association between gender and anxiety in ICD recipients. This may explain the findings since females were found to have a significantly higher somatosensory amplification than males [40]. However, Habibovic et al. [33] reported insignificant differences in anxiety levels between females and males due to the mediation effect of Type D personality.

The participants were also fearful of fatal arrhythmic deaths, shock encounters in public due to embarrassment and uncertainty of available support [14, 17, 20, 26, 44], exposure to electromagnetic interference [14, 17, 20, 23, 38], ‘cellular phone phobia’ [38, 44], ICD recalls [20], as well as driving restrictions if arrhythmias or shocks were detected [12]. There was also apprehension over resuming sexual activity as the majority feared of shocks hurting their partners [18, 20, 23, 43]. Few studies reported on the sexual concerns associated with ICD, possibly because the participants were uncomfortable in bringing up such sensitive topics with the researchers. Moreover, some became depressed over the unpredictability of their cardiac arrhythmias [30, 44] while others felt helpless over the loss of control in their lives [20, 30, 38]. Anger with one’s limitations and resentment towards ICD [15, 20, 38] were also observed. Many still harboured insecurities over the device failing or battery depleting [20, 38, 44] as well as the uncertainties that accompany arrhythmias [14] or awaiting ICD discharges [43].

Consistent across both qualitative and quantitative findings, participants with objective shocks reported more psychological distress and ICD concerns than their non-shock counterparts [16, 20, 38, 46]. Besides being reminded of their deteriorating cardiac conditions [43], participants with shock encounters ruminate of recurrences [14]. Nevertheless, they were relieved that the device functioned and had no qualms over its necessity [18, 30]. In contrast, participants without shock encounters ruminate possible future shocks [14, 17, 18, 20, 42] and at times, they continued to doubt the device [14, 18, 26]. Similar to non-shock participants, those with phantom shocks also became less trusting of the ICD as they were alarmed and confused over their reactions to future shocks [37].

#### Impacting self-identity, self-image and self-perception

ICD implantation influenced one’s body image perception [17, 20, 38]. Starrenburg et al. [35] found that females were associated with poorer device-related acceptance than males due to body image concerns. This is congruent with females’ qualitative accounts of embarrassment associated with wearing clothes that reveal their scarring [20, 26]. This may be due to greater societal expectation and emphasis on beauty in women compared to men. Moreover, according to Vazquez et al. [25], younger women tend to experience more image concerns than middle-aged and older women. Moreover, participants were conscious of the physical protrusion, arm movements, and lying down due to the awareness of the ICD in their chest [17, 42]. Some participants, however, were dissatisfied with having foreign objects inserted as it made them feel being kept alive by machines [14].

Driving restrictions also resulted in poor self-identity and self-perception where participants reported feeling ‘handicapped’, uselessness, loss of dignity, and low self-respect [12]. They viewed losing their driving license has depleted their overall well-being [30] as it is associated with the loss of independence, increasing reliance on others, and being limited in mobility and social life [12]. Nevertheless, the majority who drove before their ICD implantation had resumed driving after the restricted period [38]. Furthermore, if their license were revoked, it could have dire consequences on their employment and financial security [12, 18, 20, 30, 42].

#### Needing support and maintaining relationships

Participants with adequate support, help, and empathy from their family and social networks had better recovery and adjustments [12, 15, 20, 30, 38, 43, 47]. During the period of driving restrictions, they were transported around [30] and prevented from engaging in certain activities that were deemed risky [38]. However, not everyone was comfortable to receive help [12]. Concerns were raised regarding overprotection [15, 23, 26, 38, 43] as it made them feel dependent or being a burden [15, 18]. While some had attempted to stop their family from constantly checking on them [47], others tolerated this positively [43, 47]. By comparison, most participants felt isolated as they had lost the most important person around the time of their diagnosis and were resistant to establish new connections for fear of loss [18]. Ironically, they also emphasised the importance of independence and self-reliance to preserve self-respect [18].

Participants who feared being alone or were reluctant to go out unaccompanied [30, 38, 44] experienced reduced social activity and became dependent [38]. Being protective could also strengthen couples’ relationships [47]. Most of them became appreciative of their partners who were their pillars of support [43] and listeners in times of need [30]. However, there were also instances where reductions in sexual intimacy caused couples to become more emotionally distant [42, 43].

There was a general lack of professional support from the healthcare team [26, 30]. Nurses were viewed as knowledge experts rather than listeners or patient advocates [18]. The lack of continuity in clinical care during follow-ups reduced patients’ confidence [30] to receive support. Moreover, time constraint during follow-ups contributes to unmet emotional needs [30]. Participants also recalled encountering staff who were indifferent or uncomfortable with discussing sexual concerns [23]. Some participants accepted the uncertainty because they did not wish to bother or were unable to contact their healthcare professionals [26]. Several studies found that non-constructive support provided by healthcare professionals often led to more insecurity, psychological distress, and reduced device acceptance [30–32]. Nevertheless, there were also participants who reported receiving positive support from their healthcare team [15, 26, 30]. Such experiences varied between individuals due to potential subjectivity in how participants perceived the support based on their personal encounters.

In some studies, participants favoured joining and learning from support groups comprising of members with similar demographics and ICD experiences [14, 15, 44]. Specifically pertaining to sexual concerns, some had preferred to discuss with a support group member who is knowledgeable and experienced [23]. However, there were also others who, despite wanting to connect with ICD recipients, did not favour joining support groups [18] due to inconvenience, lack of anonymity, and on negative vibes [15, 18]. Online support chat rooms could be an alternative for these participants [15, 20].

#### Identifying learning needs

Due to the short-term inpatient stay, limited information was obtained from healthcare professionals [15, 38]. Moreover, participants were less receptive to the patient education provided in the stressful pre-ICD implantation period [12]. Although they were given resources for information [15], some still had queries [18] and were dissatisfied with the adequacy of the information provided [15, 26, 30, 38], particularly on driving restrictions and sexual concerns [12, 23]. This could potentially be due to the lack of individualised advice and information reinforcements [15, 23]. Some studies had also highlighted the lack of consistency in the information given by various healthcare professionals [12, 38]. A qualitative study by Svanholm et al. [48] revealed that some of the octogenarians were unsure if they had suffered shocks throughout their lives because of misunderstandings on shocks. Evidently, incomplete patient education could result in participants’ misinterpretations on their conditions.

A review of the articles identified 18 distinct learning needs which could be categorised into 4 main areas. These include: (1) general information on ICD where patient education on the functions, shocks, impacts, battery lifespan, and follow-ups pertaining to ICD should be given [14, 15, 23, 30, 38]; (2) diagnosis consisting of information on cardiac conditions, medications, and side effects of sudden cardiac deaths [14, 15, 18]; (3) living with ICD covers post-discharge advice on concerns like driving restrictions, resuming sexual activities, overcoming inconveniences, using electrical appliances and phones, appropriate physical activities, and swimming [12, 15, 18, 23, 38]; and (4) advanced planning for ICD deactivation [18].

#### Developing coping strategies

A cross-sectional study conducted in Sweden found that ICD recipients seldom use coping strategies and, among those used, optimism was most frequently used and highly effective [26]. Sometimes participants might have used coping mechanisms unknowingly as it occurred to them as their usual way of managing their everyday life and it had become a norm. Thus, it might not have occurred to them that these were actually also ways of coping with life after ICD implantation. Furthermore, at the moment where this study was conducted, most participants were already into their 6 to 24 months’ post-implantation and might have already adapted to the device. Thus, they would report requiring less coping strategies. A future recommendation would be to explore the coping strategies used by the participants when faced with everyday crisis prior to the implantation and compare against post-implantation findings at several time intervals to find out the changes in coping strategies as well as isolating those that are specifically used for managing ICD issues.

Most qualitative studies did not explicitly state the participants’ coping strategies and thus inference was made from their account. Several coping strategies were implicitly communicated with information belonging to subthemes like psychosocial distress or life adjustments and had to be extracted out. This review identified 12 main coping strategies which include: (1) optimistic interpretation of life situations [20, 26, 44, 47]; (2) talking about it and educating others [20]; (3) developing a strong willpower to live on [26, 44, 47]; (4) understanding own diagnosis to reduce uncertainty [14]; (5) re-evaluating outlook of life and prioritising goals [15, 20, 26, 43, 47]; (6) searching for meanings and rationalising situations [14, 37]; (7) religion and fatalism [15, 38, 43, 47]; (8) acceptance which could refer to either grateful acceptance or resigned acceptance [42]; (9) concealment of fears [42]; (10) distracting oneself with other activities and suppressing thoughts regarding diagnosis [26]; (11) evasiveness and avoidance [42–44, 47]; and lastly, (12) resignation [42, 43]. The first nine coping strategies could be considered as either neutral or adaptive while the remaining three tend to be more maladaptive. Nevertheless, such determination is subjective and dependent on one’s perception. Despite the variety of coping strategies identified, there was little information provided on its frequency and efficacy.

#### Making adjustments and gaining acceptance

Adaptations to limitations in life after ICD involves stages. In the initial period, it was about managing post-operative pain and negative emotions [15]. Most pain was experienced in the post-surgical stage and reduced thereafter [17]. Besides the surgical wound, pain was also experienced in the arm adjacent to the device due to restricted movements [42]. For the majority, such negative emotions usually dissipate after several weeks to months [20] as one learns to cope and eventually accept. Similarly, a longitudinal study by Carroll & Hamilton [45] reported improvement in the mental health score on the Medical Outcomes Study Short Form-36 (SF-36) and reduced psychological distress score on the Profile of Mood States (POMS) by 6 months’ post-implantation. Another longitudinal study by Verkerk et al. [36] also found that the mental health score on SF-36 was reduced at pre-implantation and 2 months but improved over time to be comparable with the population norm at 6 and 12 months. However, those with complications or shocks would require a longer adjustment time to build up their confidence [44].

Three qualitative findings had reported a reduction in physical functioning and activities in the initial period due to body weakness, discomfort, and reduced energy [20, 30]. These findings were consistent with the quantitative study by Verkerk et al. [36] which also reported a reduction in physical functioning score on Short Form-36 (SF-36) health survey at 2 and 6 months but improved over time to be on par with the general population at 12 months. On the contrary, Conelius [17] reported that participants actually experienced more energy and better physical functioning post-implantation. A possible explanation implied from Conelius [17] was that these participants trusted the device to protect them and were therefore more daring to engage in physical activities compared to the pre-implantation stage where they could have been more ill. However, no information was provided regarding the severity of their cardiac diagnosis and baseline physical functioning level for comparison with other studies.

Generally, participants attempted to resume their normal routine [15, 17, 20, 22, 23, 26, 44] and made adjustments to better control their lives. Some did so by placing personal restrictions and engaging in careful planning to balance activities with what was appropriate for their health [26]. Others began changing maladaptive habits to embrace a healthier lifestyle, reducing working hours to optimize life, and also avoiding activities that trigger shocks [14, 20, 26, 38]. Instead of adhering to restrictions, some participants assessed their capabilities and risks as they successively tested their limits to increase physical level [14, 26, 44].

While older adults were concerned with self-care and functioning independently [47], younger adults were more concerned with developmental transitional tasks like family planning and childbearing [20]. Some were concerned about the heredity of the cardiac conditions and decided against childbearing while those with existing children that might require ICD in future had started preparing them for it [20]. Moreover, the participants approached sexual activities more carefully by altering patterns of sexual frequency and duration [23, 43].

#### Planning for the end

A study conducted by Pedersen et al. [34] on 294 participants found that 68% of them were aware of the option for ICD deactivation or battery replacement refusal during the end-of-life whereas a smaller-scaled study on 54 participants by Raphael et al. [49] yielded only 38%. This difference could had been partially subjected to personal variations in the patient education provided by healthcare professionals. Moreover, the timing of discussing such issues also varied according to the practices of the settings where the studies were conducted as well as the patients’ conditions, ICD implantation stage, and their readiness for enhanced information. Nevertheless, the poor understanding or the lack of knowledge in ICD deactivation in both studies revealed a lack of awareness regarding end-of-life planning. Similarly, qualitative findings also reported that most of the participants interviewed expressed unawareness of the option for ICD deactivation and that some even had the misconception of equating deactivation to euthanasia [11, 48]. Furthermore, another study conducted by Stromberg et al. [29] on 3067 participants reported that only 3% had full scores on the Experiences, Attitudes, and Knowledge of End-of-Life Issues in ICD (EOL-ICD), with 29% in the 25th percentile. Notably, these findings showed that more information regarding advance planning should be given.

Insufficient knowledge on end-of-life issues often cause greater indecisiveness or making decisions that might not attain a high quality of the end-of-life years [11, 29]. Some participants had either requested for more information or expressed the willingness to be involved in such discussions with their physicians [11, 49, 50] and most had preferred to know of the options prior to their implantation [34, 49]. According to the findings in a study, the participants’ favourable attitudes towards ICD deactivation was independently associated with the avoidance of shocks during dying as they wished for a worthy and natural death [34].

## Discussion

This systematic review examined recent literature regarding the perceptions and experiences of patients living with ICD. The analysis of both quantitative and qualitative findings provided a deeper and richer insight into their quality of life, coping strategies adopted, as well as learning needs. However, caution should be exercised when interpreting these results due to the methodological limitations identified in most of the reviewed articles.

Firstly, some of the experiences recounted by the participants might inevitably be influenced by their underlying cardiac conditions, co-morbidities, and life stressors which also make up their life situations. As such, it would be difficult for participants to dissociate from other inter-related factors in their lives and share on experiences solely relating to ICD. In particular, the participants’ psychological and emotional states, as well as life adjustments, could have been partly influenced by their newly-diagnosed cardiac conditions or life-threatening encounters that warrant the ICD implantation. Secondly, it was not clearly-stated in most studies whether the participants’ ICD shock history were obtained from the medical records by researchers or participants’ self-reports. Thus, this posed a challenge in determining if the shocks described during the qualitative interviews were phantom or truly objective experiences. Despite the lack of objective measurement, phantom shocks were described with such strong conviction that they possessed a similar physical reality as objective shocks. Just as phantom limb sensations were experienced by amputees, phantom shocks experienced by ICD recipients should not be disregarded. Moreover, researchers conducting future qualitative research on objective or phantom shocks should be blinded on the participants’ shock experience so as to reduce the researchers’ influence on the participants’ account.

Ever since Kowey et al. [51] reported on the first incidence of phantom shock experience in 1992, there are still no studies in the present that has come up with a scientific account for phantom shocks. Bilanovic et al. [37] proposed a possible explanation that the participants might have perceived sub-threshold cardiac arrhythmias which fell short of being detected by the ICD as a shock therapy. This corroborated with the findings presented in another study by Kraaier et al. [52] where phantom shocks in the primary prevention group were related to a history of atrial fibrillation and potentially patients might have misinterpreted the symptoms of arrhythmia for phantom shocks. Likewise, the experiences and needs of ICD recipients with phantom shocks were also underexplored as evident by the fact that only one study published within the last 10 years was located during the systematic search. Although they belonged to a smaller subset of the ICD population, patients with phantom shocks would present different perceptions and needs. In this review, the comparison of experiences with phantom and objective shocks were limited due to the lack of published studies on phantom shocks. As such, future studies could look into exploring the perceptions of ICD recipients with phantom shock encounters. In addition, objective shocks could either be appropriate or inappropriate shock therapies delivered and since the MADIT-RIT study findings in 2012, changes to the ICD programming had reduced occurrences of inappropriate shock therapies [53]. Nevertheless, the differences in experiences among patients with appropriate and inappropriate shocks could be a potential area of future research interest. While the experiences of ICD recipients had been relatively well-explored in both quantitative and qualitative studies, and the majority of them were conducted in Western contexts. Only two of the studies were conducted in Asian settings [13, 14]. This revealed a lack of studies being conducted in Asian settings pertaining to this area where the cultural contexts can influence patients’ experiences, coping, and needs differently despite having the same implantation. It is only by examining such differences that healthcare professionals can provide more relevant and culturally-sensitive care.

In addition, there is a greater number of studies focusing on the physical, psychosocial, and emotional impacts as compared to the other domains like spiritual, socioeconomic, sexual, self-identity, and childbearing concerns. As these domains tend to involve more sensitive and close-to-the-heart issues, most participants would not freely talk about them unless raised by the researchers. Even so, some participants might be uncomfortable sharing such information with someone they had not established any rapport with. This would be a challenge especially for qualitative studies taking on a phenomenological design where a grand tour question is being posed at the beginning and the participants control the direction of the conversation till they have nothing more to say. Furthermore, there were fewer studies examining the experiences related to more specific issues like ICD recalls, end-of-life ICD deactivation, battery replacement refusal, or phantom shocks. ICD recalls refers to cases of device malfunction that would require closer monitoring rather than explanation [54]. Although these instances are rare, such experiences could be distressful and more studies are warranted in this area as well. In the recent years, there are more studies conducted to explore the experiences of patients under telemonitoring or remote home monitoring which would had implications for future practices.

## Conclusion

Although a careful systematic literature search was conducted, the search strategy may not have included all the relevant published literature. In addition, the differences in psychological impacts between appropriate and inappropriate ICD shocks may provide an interesting perspective. However, this is not included in this review as most of the articles included for this review did not differentiate between appropriate and inappropriate ICD shocks.

Nevertheless, this review indicates that ICD recipients experienced the transition from stages of uncertainty in the initial phase, to the adjustment phase, where they started to adapt and make life modifications, and finally attaining acceptance of self and trust in the ICD. It is a constant process of self-reflection, reorientation of their life perspectives, making sense of these changes, and moving on with life. Current evidence highlights the need to explore the perceptions and experiences of patients living with ICD in Asian settings.

As evident from the findings of this review, healthcare professionals tend to over-emphasise the scientific and clinical aspects rather than their patients’ actual concerns such that the lack of constructive professional support was found to inflict greater psychological distress among ICD recipients. Unlike trained healthcare professionals, most patients, being laypersons, would not be able to understand the significance of clinical results and are therefore more concerned with their quality of life and normal functioning post-implantation. This misalignment in priorities could have attributed to the dissatisfaction among ICD recipients. In order to provide good targeted care for these patients, it is pertinent for healthcare professionals to acknowledge that patients are partners in care and they have the rights to partake in the management of their own health. By listening to their patients’ concerns and daily lives, healthcare professionals could obtain a better understanding of their coping and establish therapeutic alliance to assist patients in further improving their quality of life.

## Abbreviations

ICD: Implantable cardioverter defibrillator
OS: Objective shock
PS: Phantom shock
PTSD: Post-traumatic stress disorder
QOL: Quality of life

## References

[1] Eisenberg MS (2013). Resuscitate: how your community can improve survival from sudden cardiac arrest.

[2] Mehra R (2007). Global public health problem of sudden cardiac death. J Electrocardiol.

[3] Israel CW (2014). Mechanisms of sudden cardiac death. Internal Heart J.

[4] Chugh SS, Reinier K, Teodorescu C, Evanado A, Kehr E, Samara MA (2008). Epidemiology of sudden cardiac death: clinical and research implications. Prog Cardiovasc Dis.

[5] Hayes DL, Asirvatham SJ (2007). Dictionary of cardiac pacing, defibrillation, resynchronization, and arrhythmias.

[6] Knight BP. Patient information: Implantable cardioverter-defibrillators (Beyond the basics) 2014. Retrieved from http://www.uptodate.com/contents/implantable-cardioverter-defibrillators-beyond-the-basics (accesed 10 Jan 2016).

[7] Hlatky MA, Sanders GD, Owens DK (2005). Evidence-based medicine and policy: the case of the implantable cardioverter defibrillator. Health Affair.

[8] British Medical Association Library. BMA library – Medline plus: Basic course notes for ovidSP 2012 [Booklet]. Retrieved from file:///C:/Users/Maybelline/Downloads/Medline%20Plus%20basic%20course%20manual%202012.pdf (accessed on 10 Jan 2016).

[9] The Joanna Briggs Institute (2014). Edition 2014 reviewers’ manual [Booklet].

[10] Braun V, Clarke V (2006). Using thematic analysis in psychology. Qual Res Psychol.

[11] Fluur C, Bolse K, Strömberg A, Thylén I (2013). Patients’ experiences of the implantable cardioverter defibrillator (ICD); with a focus on battery replacement and end-of-life issues. Heart Lung.

[12] Johansson I, Strömberg A (2010). Experiences of driving and driving restrictions in recipients with an implantable cardioverter defibrillator-the patient perspective. J Cardiovasc Nurs.

[13] Chair SY, Lee CK, Choi KC, Sears SF (2011). Quality of life outcomes in Chinese patients with implantable cardioverter defibrillators. Pacing Clin Electrophysiol.

[14] Saito N, Taru C, Miyawaki I (2012). Illness experience: living with arrhythmia and implantable. Kobe J Med Sci.

[15] Bolse K, Hamilton G, Flanagan Caroll DL, Fridlund B (2005). Ways of experiencing the life situation among United States patients with an implantable cardioverter defibrillator: a qualitative study. Prog Cardiovasc Nurs.

[16] Carroll DL, Hamilton GA (2005). Quality of life in implanted cardioverter defibrillator recipients: the impact of a device shock. Heart Lung.

[17] Conelius J (2015). A woman’s experience: living with an implantable cardioverter defibrillator. Appl Nurs Res.

[18] Flanagan JM, Carroll DL, Hamilton GA (2010). The long-term lived experience of patients with implantable cardioverter defibrillators. Med Surg Nurs.

[19] Groeneveld PW, Matta MA, Suh JJ, Yang F, Shea JA (2007). Quality of life among Implantable Cardioverter Defibrillator recipients in the primary prevention therapeutic era. Pacing Clin Electrophysiol.

[20] McDonough A (2009). The experiences and concerns of young adults (18–40 years) living with an implanted cardioverter defibrillator (ICD). Eur J Cardiovasc Nurs.

[21] Myers GM, James GD (2008). Social support, anxiety, and support group participation in patients with an implantable cardioverter defibrillator. Prog Cardiovasc Nurs.

[22] Salmoirago-Blotcher E, Crawford S, Tran C, Goldberg R, Rosenthal L, Ockene I (2012). Spiritual well-being may buffer psychological distress in patients with implantable cardioverter defibrillators. J Evid Based Complement Altern Med.

[23] Steinke EE, Gill-Hopple K, Valdez D, Wooster M (2005). Sexual concerns and educational needs after an implantable cardioverter defibrillator. Heart Lung.

[24] Thomas SA, Friedmann E, Gottlieb SS, Liu F, Morton PG, Chapa DW (2009). Changes in psychosocial distress in outpatients with heart failure with implantable cardioverter defibrillators. Heart Lung.

[25] Vazquez LD, Kuhl EA, Shea JB, Kirkness A, Lemon J, Whalley D (2008). Age‐specific differences in women with Implantable Cardioverter Defibrillators: an international multi center study. Pacing Clin Electrophysiol.

[26] Flemme I, Hallberg U, Johansson I, Strömberg A (2011). Uncertainty is a major concern for patients with implantable cardioverter defibrillators. Heart Lung.

[27] Flemme I, Johansson I, Strömberg A (2012). Living with life‐saving technology–coping strategies in implantable cardioverter defibrillators recipients. J Clin Nurs.

[28] Flemme I, Edvardsson N, Hinic H, Jinhage BM, Dalman M, Fridlund B (2005). Long-term quality of life and uncertainty in patients living with an implantable cardioverter defibrillator. Heart Lung.

[29] Strömberg A, Fluur C, Miller J, Chung ML, Moser DK, Thylen I (2014). ICD Recipients’ understanding of ethical issues, ICD function, and practical consequences of withdrawing the ICD in the end‐of‐life. Pacing Clin Electrophysiol.

[30] Morken IM, Severinsson E, Karlsen B (2010). Reconstructing unpredictability: experiences of living with an implantable cardioverter defibrillator over time. J Clin Nurs.

[31] Morken IM, Norekvål TM, Bru E, Larsen AI, Karlsen B (2014). Perceptions of healthcare professionals’ support, shock anxiety and device acceptance among implantable cardioverter defibrillator recipients. J Adv Nurs.

[32] Morken IM, Bru E, Norekvål TM, Larsen AI, Idsoe T, Karlsen B (2014). Perceived support from healthcare professionals, shock anxiety and post‐traumatic stress in implantable cardioverter defibrillator recipients. J Clin Nurs.

[33] Habibović M, van den Broek KC, Theuns DA, Jordaens L, Alings M, van der Voort PH (2011). Gender disparities in anxiety and quality of life in patients with an implantable cardioverter–defibrillator. Europace.

[34] Pedersen SS, Chaitsing R, Szili-Torok T, Jordaens L, Theuns DA (2013). Patients’ perspective on deactivation of the implantable cardioverter-defibrillator near the end of life. Am J Cardiol.

[35] Starrenburg A, Pedersen S, den Broek K, Kraaier K, Scholten M, Palen J (2014). Gender differences in psychological distress and quality of life in patients with an ICD 1‐Year postimplant. Pacing Clin Electrophysiol.

[36] Verkerk AJ, Vermeer AM, Smets EM, Dekker LR, Wilde AA, Van Langen IM (2015). Quality of life in young adult patients with a cardiogenetic condition receiving an ICD for primary prevention of sudden cardiac death. Pacing Clin Electrophysiol.

[37] Bilanovic A, Irvine J, Kovacs AH, Hill A, Cameron D, Katz J (2013). Uncovering phantom shocks in cardiac patients with an implantable cardioverter defibrillator. Pacing Clin Electrophysiol.

[38] Mert H, Argon G, Aslan O (2012). Experiences of patients with implantable cardioverter defibrillator in Turkey: a qualitative study. IJCS.

[39] Spindler H, Johansen JB, Andersen K, Mortensen P, Pedersen SS (2009). Gender differences in anxiety and concerns about the cardioverter defibrillator. Pacing Clin Electrophysiol.

[40] Versteeg H, Baumert J, Kolb C, Pedersen SS, Denollet J, Ronel J (2010). Somatosensory amplification mediates sex differences in psychological distress among cardioverter-defibrillator patients. Health Psychol.

[41] Colloca L, Benedetti F (2007). Nocebo hyperalgesia: how anxiety is turned into pain. Curr Opin Anaesthesiol.

[42] Humphreys NK, Lowe R, Rance J, Bennett PD (2016). Living with an implantable cardioverter defibrillator: the patients’ experience. Heart Lung.

[43] Palacios‐Ceña D, Losa ME, Salvadores‐Fuentes P, Alonso‐Blanco C, Fernández‐de‐las‐Peñas C (2011). Experience of elderly Spanish men with an implantable cardioverter‐defibrillator. Geriatr Gerontol Int.

[44] Williams AM, Young J, Nikoletti S, McRae S (2007). Getting on with life: accepting the permanency of an implantable cardioverter defibrillator. Int J Nurs Pract.

[45] Carroll DL, Hamilton GA (2008). Long-term effects of implanted cardioverter-defibrillators on health status, quality of life, and psychological state. Am J Crit Care.

[46] Jacq F, Foulldrin G, Savouré A, Anselme F, Baguelin-Pinaud A, Cribier A (2009). A comparison of anxiety, depression and quality of life between device shock and nonshock groups in implantable cardioverter defibrillator recipients. Gen Hosp Psychiat.

[47] Palacios‐Ceña D, Losa ME, Fernández‐de‐las‐Peñas C, Salvadores-Fuentes P (2011). Living with life insurance: a qualitative analysis of the experience of male implantable defibrillator recipients in Spain. J Clin Nurs.

[48] Svanholm JR, Nielsen JC, Mortensen P, Christensen CF, Birkelund R (2015). Refusing Implantable Cardioverter Defibrillator (ICD) peplacement in elderly persons—the same as giving up life: a qualitative study. Pacing Clin Electrophysiol.

[49] Raphael CE, Wing KM, Stain N, Wright I, Francis DP, Kanagaratnam P (2011). Implantable cardioverter defibrillator recipient attitudes towards device deactivation: how much do patients want to know?. Pacing Clin Electrophysiol.

[50] Herman D, Stros P, Curila K, Kebza V, Osmancik P (2013). Deactivation of implantable cardioverter-defibrillators: results of patient surveys. Europace.

[51] Kowey PR, Marinchak RA, Rials SJ (1992). Things that go bang in the night. N Engl J Med.

[52] Kraaier K, Starrenburg A, Verheggen R, Van der Palen J, Scholten M (2013). Incidence and predictors of phantom shocks in implantable cardioverter defibrillator recipients. Neth Heart J.

[53] Moss AJ, Schuger C, Beck CA, Brown MW, Cannom DS, Daubert JP (2013). Reduction in inappropriate therapy and mortality through ICD programming. N Engl J Med.

[54] Kirian KB, Sears SF, Shea JB (2009). How to respond to an implantable cardioverter-defibrillator recall. Circulation.

